# Highly Sensitive and Accurate Assessment of Minimal Residual Disease in Chronic Lymphocytic Leukemia Using the Novel CD160-ROR1 Assay

**DOI:** 10.3389/fonc.2020.597730

**Published:** 2020-12-03

**Authors:** Timothy W. Farren, Kaushik S. Sadanand, Samir G. Agrawal

**Affiliations:** ^1^ Department of Haemato-Oncology and Immunophenotyping (SIHMDS), Barts Health NHS Trust, London, United Kingdom; ^2^ Immunobiology, Blizard Institute, Queen Mary University of London, London, United Kingdom

**Keywords:** CD160, ROR-1, minimal/measurable residual disease, chronic lymphocytic leukemia, monoclonal B-cell lymphocytosis, flow cytometry, immunophenotyping

## Abstract

Undetectable minimal residual disease (MRD) in Chronic Lymphocytic Leukemia (CLL) has a favorable prognostic outcome compared with MRD that can be detected. This study investigated a flow cytometric assay (CD160-ROR1FCA) targeting the tumor-specific antigens CD160 and receptor tyrosine kinase-like orphan receptor 1 (ROR1), along with CD2, CD5, CD19, CD45. CD160-ROR1FCA was compared with the originally published 8-colour European Research Initiative for CLL (ERIC) gold-standard assay for CLL MRD detection. CD160-ROR1FCA had a limit of detection of 0.001% and showed strong correlation with ERIC (*R* = 0.98, p < 0.01) with negligible differences in MRD detection (bias -0.3152 95%CI 5.586 to -6.216). Using CD160-ROR1FCA, increased expression of both CD160 and ROR1 was found in Monoclonal B cell Lymphocytosis (MBL) compared to low-level polyclonal B-cell expansions (p < 0.01). Patients in CR and with undetectable MRD had a longer EFS (not reached) than those in CR but with detectable MRD (756 days, p < 0.01) versus 113 days in patients with partial remission (p < 0.01). Patients with MRD levels of >0.01 to 0.1% had a longer EFS (2,333 days), versus levels between 0.1 to 1% (1,049 days). CD160-ROR1FCA is a novel assay for routine CLL MRD measurement and for MBL detection. MRD status assessed by CD160-ROR1FCA after CLL treatment correlated with EFS.

## Introduction

Over the last two decades, there has been a paradigm shift in the treatment of patients with chronic lymphocytic leukemia (CLL), which has led to the ability to achieve a deep minimal/measurable residual disease (MRD) undetectable remission. For many years, palliation was the norm using monotherapy, such as chlorambucil (1), which evolved to combination chemotherapy and then chemo-immunotherapy (2). The Fludarabine, Cyclophosphamide, Rituximab (FCR) regimen led to higher complete remission (CR) rates than seen before and, importantly, undetectable MRD CR (3). More recently, CLL management has been transformed again with the use of tyrosine kinase inhibitors (TKI) targeting the B-cell receptor (BCR) signaling pathway, such as Ibrutinib (4, 5) and Idelalisib (6), and the Bcl-2 inhibitor, Venetoclax (7). The greater understanding of the molecular biology of CLL has revealed pre-treatment factors, such as deletions of 17p or certain genetic mutations, that can predict the post treatment risk of relapse and these have been key drivers for the use of more personalized targeted therapy (8, 9).

The development of these therapies has made CR in CLL a realistic endpoint in many clinical trials. However, achieving a CR does not necessarily equate to undetectable MRD. MRD means that tumor cells cannot be detected by standard morphological diagnostic methods, but only with more sensitive methods, such as allele-specific PCR or flow cytometry (10). The accurate quantitation of residual disease burden following therapy is now an established technique for several hematological malignancies, including acute lymphoblastic leukemia (ALL) (11), myeloma (12) and CLL (13). Undetectable MRD has been shown to demonstrate prognostic significance both in terms of progression free survival (PFS) and overall survival (OS) independent of prognostic risk stratification or choice of therapy (14). Furthermore, not only is the detection of MRD of significance, but the level of detectable disease is known to influence prognosis, with a threshold greater than 0.01% (10^−4^) proving to be an independent predictor of PFS and OS in patients with CLL treated with chemo-immunotherapy (14–16). However, the role of MRD assessment with novel cellular immunotherapies, such as Bi-specific T-cell engagers (BiTEs) and Chimeric Antigen Receptor (CAR) T cells, is yet to be defined.

The need to determine residual disease with high sensitivity and specificity, has led to the search for tumor specific antigens, or leukemia-associated immunophenotypes (LAIPs) that differ from the majority of normal hematopoietic cells in order to simplify the detection of MRD (17). The Natural Killer (NK)-cell-activating receptor, CD160, is aberrantly expressed by CLL cells and other B-cell malignancies, but is not expressed by normal B-cells, making it a good target both diagnostically and for MRD analysis in CLL (18, 19). CD160 is an 80kDa immunoglobulin (Ig)-like glycoprotein cell surface receptor anchored to glycosylphosphatidylinositol (GPI) (20). It is normally found on CD56^dim^ CD16^+^ cytotoxic NK cells (20, 21), cytotoxic CD8+ (TCRαβ) T lymphocytes (22), TCRγδ T cells (20) and intestinal intraepithelial lymphocytes (iIELs) (23). CD160 is also a T cell “exhaustion” marker (24) and expressed on neo-angiogenic endothelial cells in solid tumors (25). CD160 may be implicated in the pathophysiology of CLL by enhancing cell activation, survival and cytokine release *via* the PI3k pathway (18, 26, 27).

Combining multiple tumor specific antigens into one assay provides a simplified yet highly specific approach to MRD analysis. Another tumor specific antigen for malignant B-cells is the cell surface tyrosine kinase-like orphan receptor 1 (ROR1) (28–31). Like CD160, ROR1 has been utilized diagnostically (32) and has shown to be constitutively phosphorylated in CLL (33).

ROR proteins are prominently expressed in embryogenesis and are evolutionarily conserved (34). ROR1 plays a role in carcinogenesis and embryogenesis through its close resemblance to the tropomyosin receptor kinase (Trk family) neurotropic receptors and association with the Wnt-family signaling proteins *via* its cysteine-rich domain shared with Frizzled receptors (28, 34). Non-malignant peripheral blood lymphocytes do not normally express ROR1 (35). With the ROR proteins being associated to the Wnt receptor family, specifically interacting with the WNT5a pathway with downstream activation of NF-kB in B cell malignancies, there is increasing evidence that aberrant ROR1 expression contributes to a number of hematological malignancies (32). Specifically in B-CLL, ROR1 expression plays a pivotal role in cell survival by inhibiting apoptosis of the malignant B-cells *via* the activation of PI3K/AKT/mTOR and MEK/ERK pathways (36). Downregulation of the ROR1 protein and silencing the ROR1 gene abrogates this pathway and enables apoptosis of CLL cells (36–39).

The European Research Initiative on CLL (ERIC) has previously published several reports on the harmonization of MRD assessment using both 4-color and 6-color panels (15, 40).

The aim of this study was to investigate the utility of combining the tumor specific antigen, CD160, and the tumor associated antigen, ROR1, into a new single tube flow cytometric assay (CD160-ROR1FCA) for highly sensitive detection of measurable disease in CLL. This assay was validated against previously published data utilizing the CD160 flow cytometric assay (CD160FCA) (18, 19) and the single tube 8-color panel designed by the ERIC group, considered as the original “gold standard” for CLL MRD detection (41, 42).

## Patients and Methods

### Patient Samples

Between October 2012 and February 2014, prospective assessment of MRD status was performed on peripheral blood of patients with CLL. The diagnosis of CLL was based on the International Workshop on CLL (43) and BCSH guidelines (44). Leukocytes were prepared from a total of 140 samples from 89 patients with CLL. Normal donor controls were sourced from anonymised peripheral blood samples undergoing investigation for a non-hematological related assessment or reactive lymphocytosis. Informed consent for sample collection was obtained in all cases. A total of 13 patients diagnosed with monoclonal B-cell lymphocytosis were recruited into the study. The NHS Health Research Authority, National Research Ethics Service at Westminster London approved non-diagnostic analysis, and written informed consent was obtained (REC reference 07/Q0604/34 and REC reference 13/LO/0284, IRAS ID: 105378).

### Simulation of MRD

For the assessment of proof of concept of the CD160-ROR1FCA, and to confirm the limit of detection (LoD) and limit of quantification (LoQ), 5 samples from patients with typical immunophenotype of CLL (Score 5/5), were serially diluted five times with 1:10 dilutions into normal leucocytes. It has already been established that the minimum discrete cluster population size for CLL MRD detection is 20 events. The limit of detection is therefore defined as 20/total nucleated cells (TNCs, 200,000 TNCs to reach a LoD of 0.01%). For this study, reliable quantification of CLL MRD required 50 clustered events, thus defining the limit of quantification (LoQ) as 50/TNCs. Therefore to reach a LoQ, 500,000 TNCs were required to reach a LoQ of 0.01%. By using normal leucocytes and collecting enough cells, it was possible to demonstrate an LoQ of 0.003%.

### CD160-ROR1 Flow Cytometric Assay for MRD Detection in Patients

The CD160FCA has been previously reported for use diagnostically and to detect residual disease (18, 19). The CD160FCA panel was modified to include ROR1 (28). For the development of the core marker CD160-ROR1 panel, leukocytes were prepared by ammonium chloride lysis from six patients with CLL at presentation and each diluted into normal leukocytes in five serial 1:10 dilutions.

The CD160-ROR1 assay incorporated CD2 (Clone S5.2), CD5 (L17F12), CD19 (Clone SJ25C1), CD23 (Clone EBVCS-5), and CD45 (Clone Hi30) monoclonal antibodies from BD Biosciences, Oxford, UK. ROR-1 (Clone 4A5), and CD160 (Clone BY55) were obtained directly from BD Biosciences, San Diego, USA. Together these formed the “CD160-ROR1FCA”. Identification of residual disease populations used a validated sequential gating strategy for the detection of CD160 and ROR-1 co-expression on malignant CD19+ B cells. Initial gating focused on CD45 positive events versus side scatter, followed by forward and side scatter to gate the lymphoid region and exclude any apoptotic cells and debris. Total B cells were identified using CD19+ B cells were compared with side scatter to exclude any nonspecific binding. The CD19+ B cells were further isolated by gating the CD2+ events and generating a Boolean NOT exclusion gate. The malignant B cells were separated from the normal residual B cells using a CD45+ CD2- CD5+ CD19+ CD23+ gate, with subsequent gating on both CD160+ and ROR-1 positivity. The presence of residual disease was defined on the number of events co-expressing CD45+/CD2-/CD5+/CD19+/CD23+/ROR-1+/CD160+, out of the total CD45+ total nucleated cell population (**Supplemental Figure 1**).

The CD160-ROR1FCA was compared with CD160FCA and the 8-color ERIC consortium protocol.

The 8-color ERIC panel consisted of 8 antibodies targeting CD3 (V500-C), CD5 (V450), CD19 (Pe-Cy7), CD20 (APC-H7), CD22 (PerCPCy5.5), CD43 (APC), CD79b (PE), and CD81 (FITC). It is currently the most accurate technique for MRD assessment in CLL by flow cytometry, and has provided a complementary role to high-throughput sequencing of patients with CLL (41, 42).

All monoclonal antibodies used in the study underwent titration to determine the optimum concentration of monoclonal antibody for a given number of cells. The assay also underwent steric hindrance analysis for targeting macromolecular complexes. The intrinsic spectral overlap of the different fluorochromes in the CD160-ROR1FCA was corrected using compensation matrices and automatic compensation beads (BD Biosciences). A sequential gating strategy was used.

A minimum of two million leukocytes were incubated for both MRD panels using the ammonium chloride based approach as previously described (18, 19). To achieve a sensitivity of 10^-4^, a minimum of >500,000 events were acquired on a FACS Canto II with standard laser and filter configuration (BD Biosciences). The aim was to achieve 2.0x10^6^ total nucleated cells to reach a level of 10^-5^. Light chain analysis (LCR) was performed in all cases and reported where detectable. Data was analyzed using the BD FACS Diva clinical software (version 6.1.3) for enhanced acquisition analysis, which determined the median fluorescence intensity (MFI) of CD160 and ROR1 on the CD2-negative CD5+CD19+CD23+ population.

### Statistical Methods

Standard epidemiological approaches were used to calculate diagnostic indices of sensitivity and specificity. To determine non-random associations, the two-tailed nonparametric Fisher’s exact test was used. Nonparametric correlation coefficient r (Spearman Rank) was calculated to compare the MRD results obtained from the different centers. To determine differences in MFI of MRD populations, the Mann-Whitney two-tailed t-test based on the mean ± S.E.M. was used.

Bland–Altman plots, mean difference (AVERAGE function) and 95% limit of agreement, reported as ± 1.96 s.d (STDEV function), were calculated from log-transformed data. The level of significance was set at <0.05%. Statistical analysis was performed using GraphPad PRISM 5.0d for Macintosh (GraphPad software, CA, USA) and SPSS Statistics for Macintosh, Version 22.0 (Armonk, NY: IBM Corp).

## Results

### Assessment of Core Markers for Reproducible Detection of MRD in CLL

The assays assessed for residual disease all show a disease-specific expression to CLL. The CD160FCA (CD19, CD5, CD23, CD45, CD160, and CD2), CD160-ROR1FCA (CD19, CD5, CD23, CD45, CD160, ROR1, and CD2) and ERIC methods (CD19, CD20, CD5, CD43, CD79b, CD81, CD22, and CD3) all detected a high percentage of disease-specific antigen expression on the lymphoid population of patients with CLL, and a low percentage expression on both CLL-like and non-CLL MBL cases (**Supplemental Figure 2**; p < 0.01).

### Spiking Experiments to Simulate MRD

**Figure 1:**
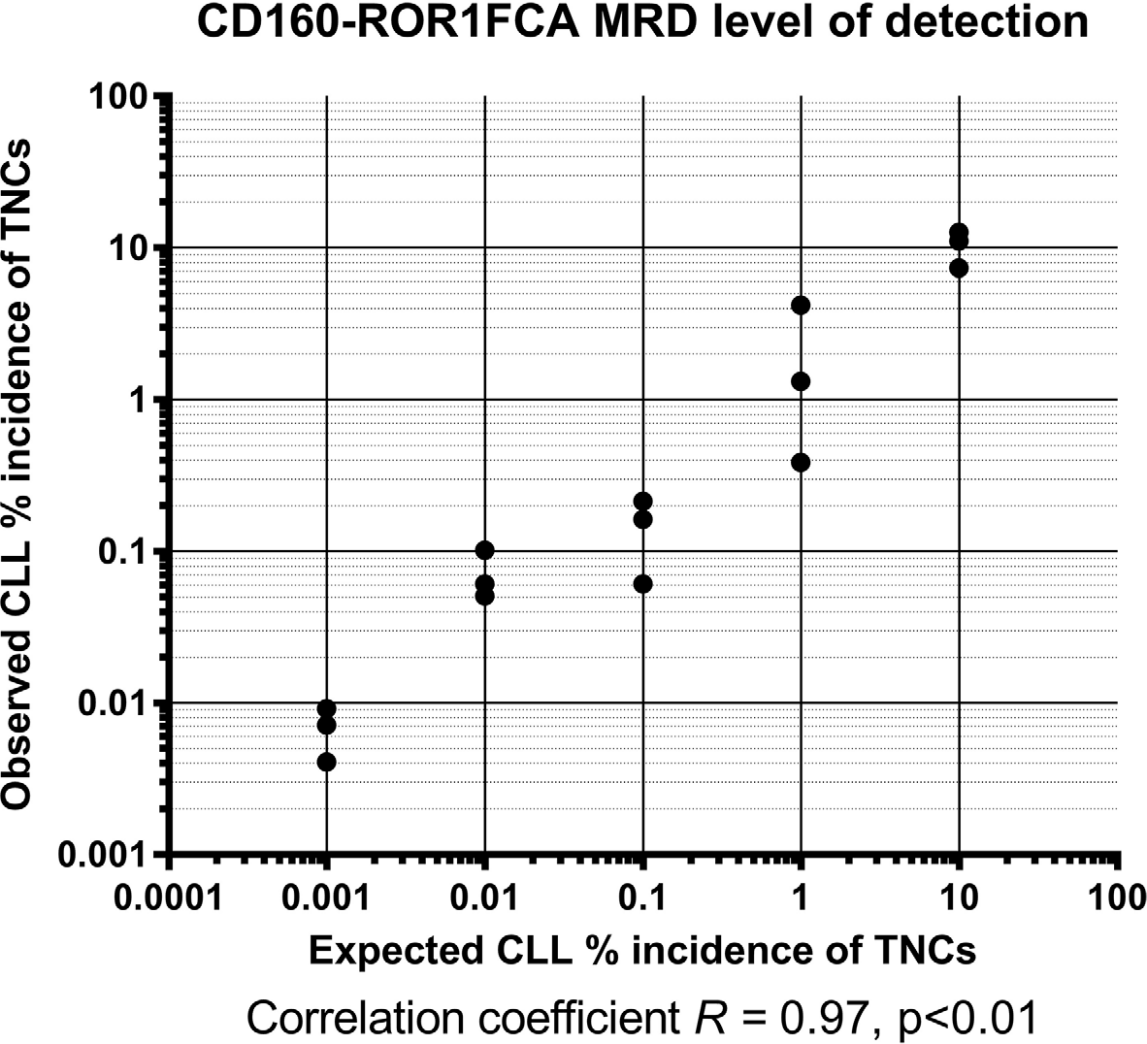
CD160-ROR1FCA MRD level of detection. Samples from three patients with CLL were serially diluted in normal leukocytes, such that the CLL cells represented an actual tumor burden as low as 0.001% of the total nucleated cell population. The observed MRD detection with CD160-ROR1FCA showed excellent concordance with the theoretical MRD level: limit of detection 0.001%, with a limit of reportable quantification of 0.003% (**Figure 1**, R^2^ = 0.978, p < 0.01).

The feasibility and validation of using the CD160-ROR1FCA for MRD detection was assessed using a simulation assay. Samples from three patients with CLL were serially diluted in normal leukocytes, such that the CLL cells represented an actual tumor burden as low as 0.001% of the total nucleated cell population. When observing theoretical MRD detection against observed in these spiking experiments, there was a highly significant degree of concordance between the two, with an established LoD level of 0.001%, although this dataset had a limit of reportable quantification of 0.003% (**Figure 1**, R^2^ = 0.978, p < 0.01). Assessment of the observed incidence against expected incidence of CLL MRD demonstrated a highly significant correlation between the CD160-ROR1FCA and ERIC assays throughout the spiking experiments, confirming the proof of concept for both (data not shown).

### Comparison of Sensitivity Between CD160-ROR1FCA With CD160FCA and ERIC

Having previously demonstrated that the CD160FCA can reliably detect residual CLL to a threshold of 10^-4^ (0.01%) (19), in this study, the CD160-ROR1FCA was validated against the original CD160FCA and ERIC methodologies down to an accuracy of 10^-5^ (0.001%). Where possible surface light chain expression was also correlated. MRD results were reported as a percentage of the absolute number of total nucleated cells.

**Figure 2:**
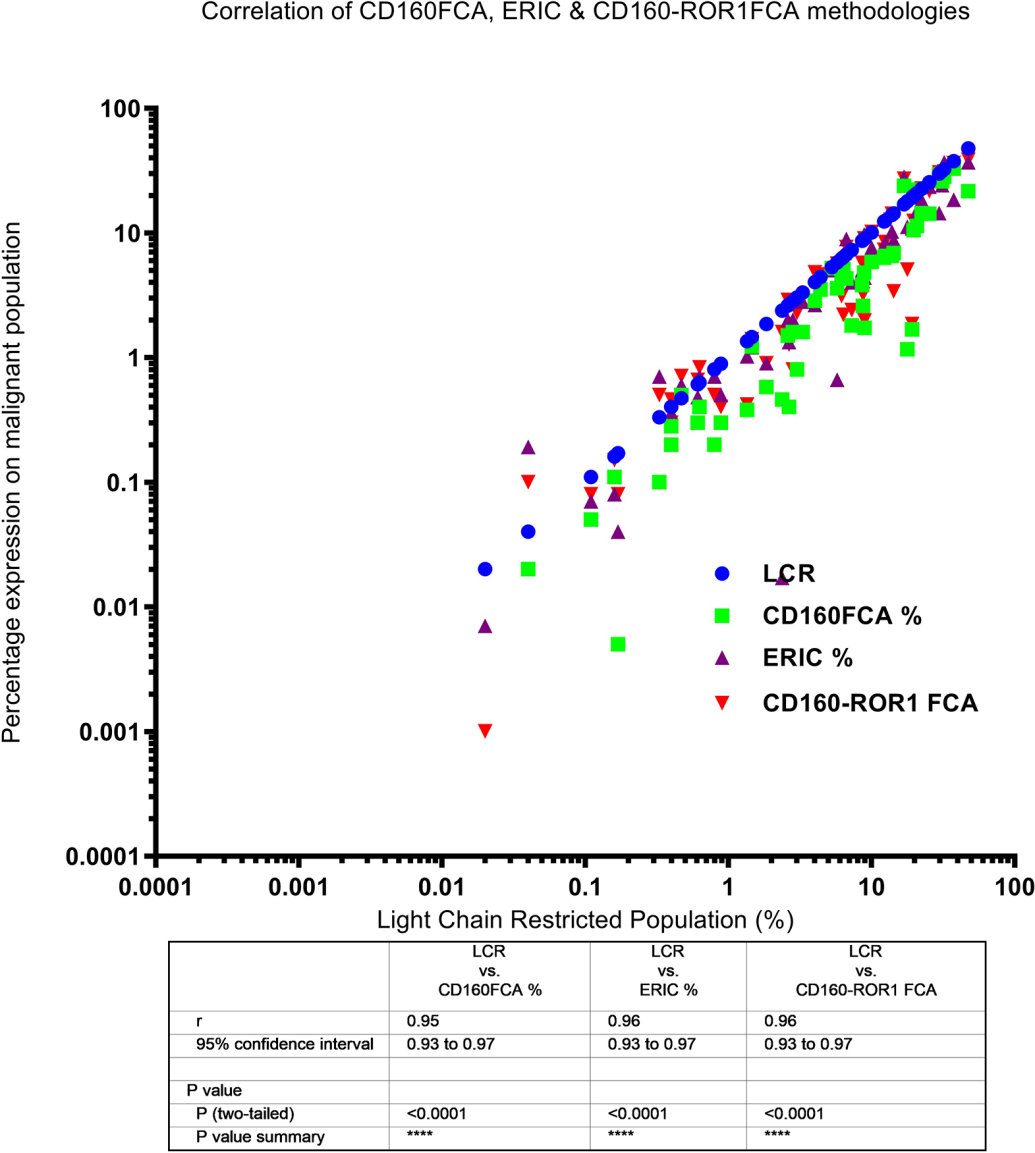
Correlation of light chain restriction and MRD detection by CD160FCA, the ERIC protocol and CD160-ROR1FCA. Light chain restriction (LCR, shown as blue dots) was detectable in 24 patients. The percentage MRD expression is plotted for each of the assays against the percentage of the LCR-restricted population: CD160FCA – green squares; ERIC – purple triangles; CD160-ROR1FCA – red triangles. There was excellent correlation between level of LCR and detectable disease with all the assays (p < 0.01).

Light chain restriction (LCR) was detectable in 24 patients (size of the restricted population ranged from 0.2 to 47% of all cellular events). This sub-group of patients also demonstrated excellent correlation between level of LCR and detectable disease by CD160FCA (Spearman R = 0.95, 95%CI: 0.93–0.97, p < 0.0001), ERIC (R = 0.96, 95%CI: 0.93–0.97, p < 0.0001) and CD160-ROR1FCA (R = 0.96, 95%CI 0.93–0.97, p < 0.0001) (**Figure 2**).

**Figure 3:**
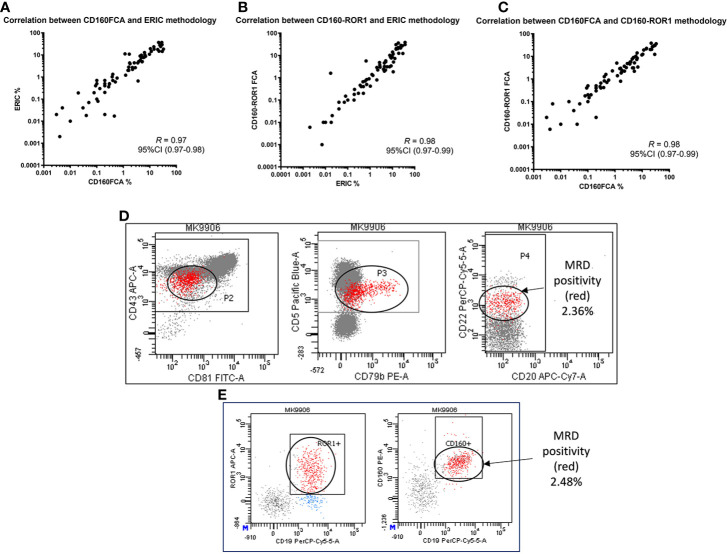
Correlation of MRD detection by CD160FCA, the ERIC protocol and CD160-ROR1FCA. Patient samples (n = 89) were simultaneously assessed for MRD using the three assays. **(A)** CD160FCA vs ERIC (correlation coefficient of *R* = 0.97, p < 0.01). **(B)** ERIC and CD160-ROR1FCA (*R* = 0.98, p < 0.01). **(C)** CD160FCA and CD160-ROR1FCA (*R* = 0.98, p < 0.01). All MRD results were reported following clear gating on the aberrantly expressed population. **(D)** Patient MK with identifiable disease (red population) by ERIC methodology accounting for 2.36% of TNCs. **(E)** Patient MK with identifiable disease (red population) by CD160-ROR1FCA methodology, accounting for 2.48% of TNCs.

In this series, there was high concordance between all three assays for the assessment of MRD. The original reported CD160FCA *vs* ERIC 8-color assay demonstrated a correlation coefficient of *R* = 0.97 (95%CI: 0.97–0.98, p < 0.01, **Figure 3A**). The correlation coefficient between the ERIC and CD160-ROR1FCA was *R* = 0.98, p <0.01 (**Figure 3B**), while for CD160FCA and CD160-ROR1FCA, *R* = 0.98, p <0.01 (**Figure 3C**). All MRD results were reported following clear gating on the aberrantly expressed population. Both the ERIC and CD160-ROR1FCA generated clearly identifiable and comparable residual populations (**Figures 3D, E** respectively).

When determining quantifiable disease at levels between 0.01–1%, both CD160-ROR1FCA and ERIC assays have a smaller difference in detection between each method, than at higher levels of disease (>1%). For all levels of disease (as a percentage), the difference in residual disease detection between the two methods is still small (bias -0.3152 95%CI 5.586 to -6.216), thus the two assays are very comparable (Figure not included).

**Figure 4:**
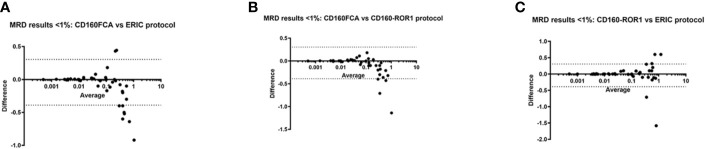
Differences in MRD detection by CD160FCA, the ERIC protocol and CD160-ROR1FCA. Patient samples (n = 54/89) with MRD detection at low levels (< 1%) were subject to Bland-Altman comparison for differences in MRD detection. **(A)** CD160FCA *vs* ERIC: Bias: -0.04 (95%CI -0.39 to 0.30. **(B)** CD160FCA v CD160-ROR1 Bias: -0.05 (95%CI -0.37 to 0.28). **(C)** ERIC v CD160-ROR1 Bias: -0.002 (95%CI -0.42 to 0.41).

Restricting analysis to low MRD at a level <1%, 54/89 patients had quantifiable disease. Bland-Altman assay comparison in this dataset demonstrated significant associations between all three assays. CD160FCA *vs* ERIC: Bias: -0.04 (95%CI -0.39 to 0.30, **Figure 4A**); CD160FCA v CD160-ROR1 Bias: -0.05 (95%CI -0.37 to 0.28, **Figure 4B**); ERIC v CD160-ROR1 Bias: -0.002 (95%CI -0.42 to 0.41, **Figure 4C**)

### Assessment of ROR1 and CD160 in MBL

**Figure 5:**
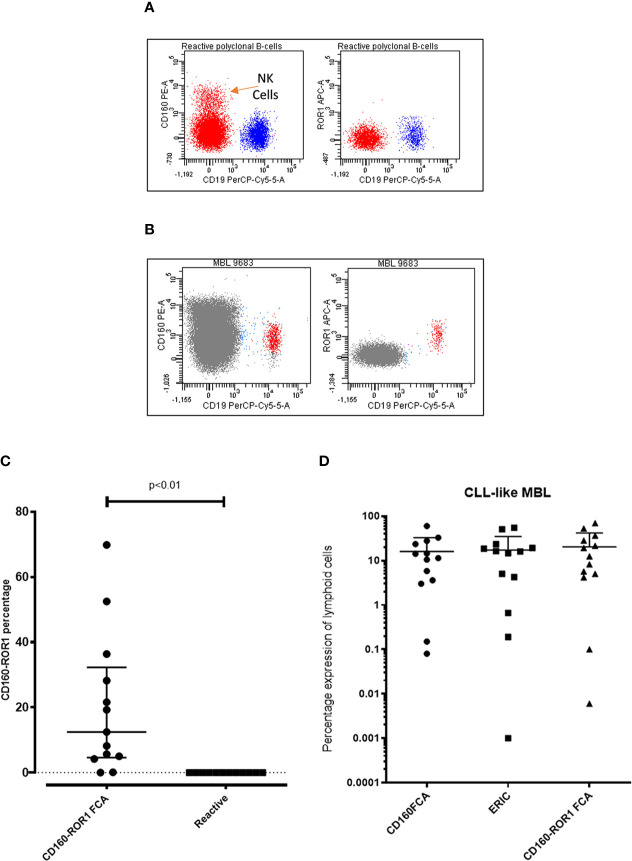
Assessment of ROR1 and CD160 in Monoclonal B-cell lymphocytosis. Patient samples with monoclonal B-cell lymphocytosis (MBL, n = 13) were simultaneously assessed by CD160FCA, the ERIC protocol and CD160-ROR1FCA. **(A)** Dot plot demonstrating CD160 and ROR-1 negativity on polyclonal B-cells from a reactive case. **(B)** Dot plot demonstrating CD160 and ROR-1 positivity on a case of monoclonal B-cell lymphocytosis. **(C)** CD160-ROR1FCA percentage expression in MBL versus samples with polyclonal B-cells (“Reactive”, n = 13, p < 0.01). **(D)** Comparison of the three assays in CLL-like MBL (n = 13).

The application of both ROR1 and CD160 in the diagnostic setting as well as measurable disease analysis post therapy has been clearly demonstrated (18, 19, 32). The application of CD160-ROR1FCA was assessed in the context of monoclonal B-cell lymphocytosis (MBL), a well established “pre-leukemic” phase to CLL. Previous investigations have shown CD160 to be expressed in MBL (18). ROR-1 was also found to be expressed in MBL (n = 13), with similar expression observed in CLL. Detection of the MBL populations (**Figure 5B**) against polyclonal B-cells (**Figure 5A**) from patients with low level lymphocytosis undergoing investigation for non-hematological conditions, showed a significant upregulation of both proteins suggesting CD160 and ROR1 are both targets for pre-leukemic MBL (p < 0.01, **Figure 5C**). Comparison between all three methodologies demonstrated a strong concordance regardless of analysis used: CD160FCA *vs* ERIC *R* = 0.85); ERIC vs CD160-ROR1 *R* = 0.91; CD160FCA v CD160-ROR1 *R* = 0.96 (**Figure 5D**).

### Undetectable MRD as a Potential Surrogate for EFS

**Figure 6:**
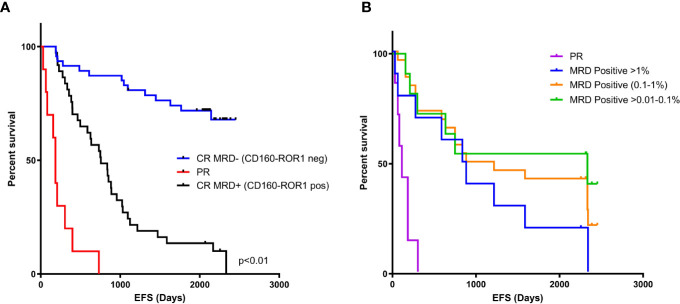
MRD negativity by CD160-ROR1FCA correlates with event-free survival. Event-free survival (EFS) was assessed against MRD status measured by CD160-ROR1FCA. **(A)** EFS of patients in CR and MRD negative <0.01% (blue line, days not reached), CR and MRD positive >0.01% (black line, 756 days p < 0.01) and patients achieving a PR (red line, 113 days, p < 0.01). **(B)** Level of MRD and EFS: >0.01 to 0.1% (green line) 2,333 days versus 0.1 to 1% (orange line) 1,049 days (p = 0.037).

Measurable residual disease <0.01% for patients in complete remission (CR) using detectable ROR1/CD160, are considered as true MRD negative. The event free survival for this group had yet to be reached in this cohort (**Figure 6A**). Those patients in CR, but with detectable MRD (> 0.01%) had a median EFS of 756 days (p < 0.01), and those with partial remission of 113 days (p < 0.01). The level of MRD correlated with EFS as those patients with >0.01 to 0.1% residual disease the EFS was 2,333 days, versus 1,049 days for those patients with MRD between 0.1 to 1% (**Figure 6B**, p = 0.037).

Measurable residual disease below 0.01% (0.001–0.01%), but not validated as quantifiable, had no significant difference in terms of EFS when compared with 0.01–0.1% MRD (data not shown).

## Discussion

There has been a dramatic shift in the treatment of CLL, with the introduction of combination chemo-immunotherapy and, more recently, TKIs and Venetoclax, to achieve a CR (2–7). Despite the achievement of a CR, there may be residual CLL cells, detectable only by more sensitive methods of PCR and flow cytometry. It has been shown that patients in CR with undetectable MRD have a superior prognosis with an enhanced PFS and OS than those patients with detectable MRD (13, 14, 19). This study evaluates the utility of a novel flow cytometric assay for MRD detection in CLL, the CD160-ROR1FCA. By combining two tumor-specific markers of the B-cell lineage, CD160 and ROR-1 (18, 19, 30, 32, 45), CD160-ROR1FCA targets fewer CLL surface antigens than the originally published ERIC methodology, thereby reducing the number of monoclonal antibodies used, and allowed a simpler gating strategy and remains highly sensitive. Since its initial publication, the ERIC methodology has proposed dropping the CD3 and CD22 from the panel, leading to a six-color panel that is widely utilized.

In this study, the CD160-ROR1FCA was compared against the original 8-colour gold-standard ERIC method (41, 42). The data in this study were obtained from patients that were managed at Barts Health NHS Trust, regardless of their CLL treatment regimen. MRD assessment was performed in the diagnostic immunophenotyping laboratory and undetectable MRD was defined as <0.01% (10^-4^; i.e. less than 1 malignant cell in 10,000 normal peripheral blood cells). Both the CD160-ROR1FCA and 8-color ERIC methodologies detected MRD to a level of <0.01% (10^-5^; that is, less than 1 malignant cell in 100,000 normal PB cells). Comparison of the new CD160-ROR1FCA with the original gold standard, ERIC method, showed a high level of correlation and concordance (p < 0.0001). When observing MRD below 1%, the original CD160FCA was the least sensitive for quantifiable MRD, but there was no statistically significant difference between CD160-ROR1FCA and ERIC.

In previous work, using the CD160FCA assay, the labile nature of CD160 marker expression meant results from samples older than 48 h needed to be interpreted with caution (12). The addition of ROR-1 targeting has helped to eliminate this problem. We also reported that the CD160FCA could quantify MRD to the same level of sensitivity (> 0.01%) in both peripheral blood (PB) and bone marrow (BM) (19). This study has demonstrated that all three methods produce highly concordant results, and whilst the original CD160FCA produced the greatest variation, it was not clinically significant. One limitation of the CD160-ROR1FCA is that only peripheral blood samples can be assessed, as ROR1 is expressed on a subset of haematogones, a non-neoplastic precursor stage of B cells, found within the bone marrow (35). This can be resolved by separating the CD160 and ROR1 signals by using different fluorochromes for the antibodies, rather than combining them as in this study, as CD160 is not present on haematogones.

Level of measurable residual disease using the ROR1/CD160 combination correlated with event free survival. In those patients with ≥0.1% MRD following therapy, the time to intervention was significantly shorter than 0.01–<0.1% (p = 0.034). Those patients with >1% MRD had significantly shorter time to intervention over the lower log reductions in detectable disease, but significantly improved over those patients who only achieved a partial remission (p = 0.02). Utilizing the validated minimum phenotype cluster of 20 events in at least 200,000 leucocytes generated the reproducible limit of detection of 0.01%, while 50 cells clustering in 500,000 leucocytes generated the Limit of Quantification of 0.001%. The addition of the CD3 in the ERIC method allowed quantifiable detectable levels below the reportable LoQ, which permitted the CD160-ROR1FCA to be validated against this reference methodology with a strong correlation.

Monitoring MRD in CLL is a key focus for clinical trials, as MRD is an important prognostic marker for PFS and OS (14). The assessment of MRD status for CLL is likely to become routine in clinical practice, as MRD status starts to impact on patient management. However, further research is needed to determine whether MRD status can determine the duration of treatment or guide a change in therapy. Of interest in this context, is the use of MRD to potentially shorten the standard 6 cycles of FCR (Fludarabine/Cyclophosphamide/Rituximab) chemo-immunotherapy based on early achievement of MRD negativity (16, 46), with the goal of reducing treatment-related toxicity. The role of MRD assessment is also unknown in the context of the targeted therapies. The design of the MURANO study (47), with a fixed duration of treatment with Venetoclax (24 months) in combination with Rituximab, highlights this issue—should patients who remain MRD positive continue treatment beyond 24 months? Can early achievers of MRD negativity stop treatment before 24 months? In addition to reducing treatment-related toxicity, there is significant impact of reducing the financial burden of prolonged therapy with Venetoclax and TKIs (48).

The CD160-ROR1FCA, similar to the modified 6-color ERIC methodology, is a rapid, highly sensitive, single tube assay with a simple gating strategy. The unique nature of having two tumor-specific targets for the characterization of residual tumor cells could be applied to any flow cytometric assay, which could be particularly helpful for the evaluation of novel therapies including BiTE and CAR-T cell trials.

## Data Availability Statement

The original contributions presented in the study are included in the article/**Supplementary Materials**. Further inquiries can be directed to the corresponding author.

## Ethics Statement

The studies involving human participants were reviewed and approved by NHS Health Research Authority, National Research Ethics Service. The patients/participants provided their written informed consent to participate in this study.

## Author Contributions

TF and SA designed experiments as well as analyzed the data. TF and KS performed experiments. SA designed and supervised the experiments. TF and SA provided funding for the experiments. TF, KS, and SA wrote the paper. Sections of this paper were submitted as part of KS’s BSc Dissertation in 2018 at Queen Mary University of London. All authors contributed to the article and approved the submitted version.

## Funding

The authors declare that this study received funding and reagents listed within the manuscript from BD Biosciences (San Jose, CA). The funder was not involved in the study design, collection, analysis, interpretation of data, the writing of this article or the decision to submit it for publication.

## Conflict of Interest

The authors declare that the research was conducted in the absence of any commercial or financial relationships that could be construed as a potential conflict of interest.
