# Supplementary material for: Highly Sensitive and Accurate Assessment of Minimal Residual Disease in Chronic Lymphocytic Leukemia Using the Novel CD160-ROR1 Assay
Source: Front Oncol. 2020 Dec 3;10:597730. doi: 10.3389/fonc.2020.597730 (PMC7744938; doi:10.3389/fonc.2020.597730)
Supplement: Supplementary file 1 [file Table_1.docx]

Supplementary Material

## Supplementary Figures


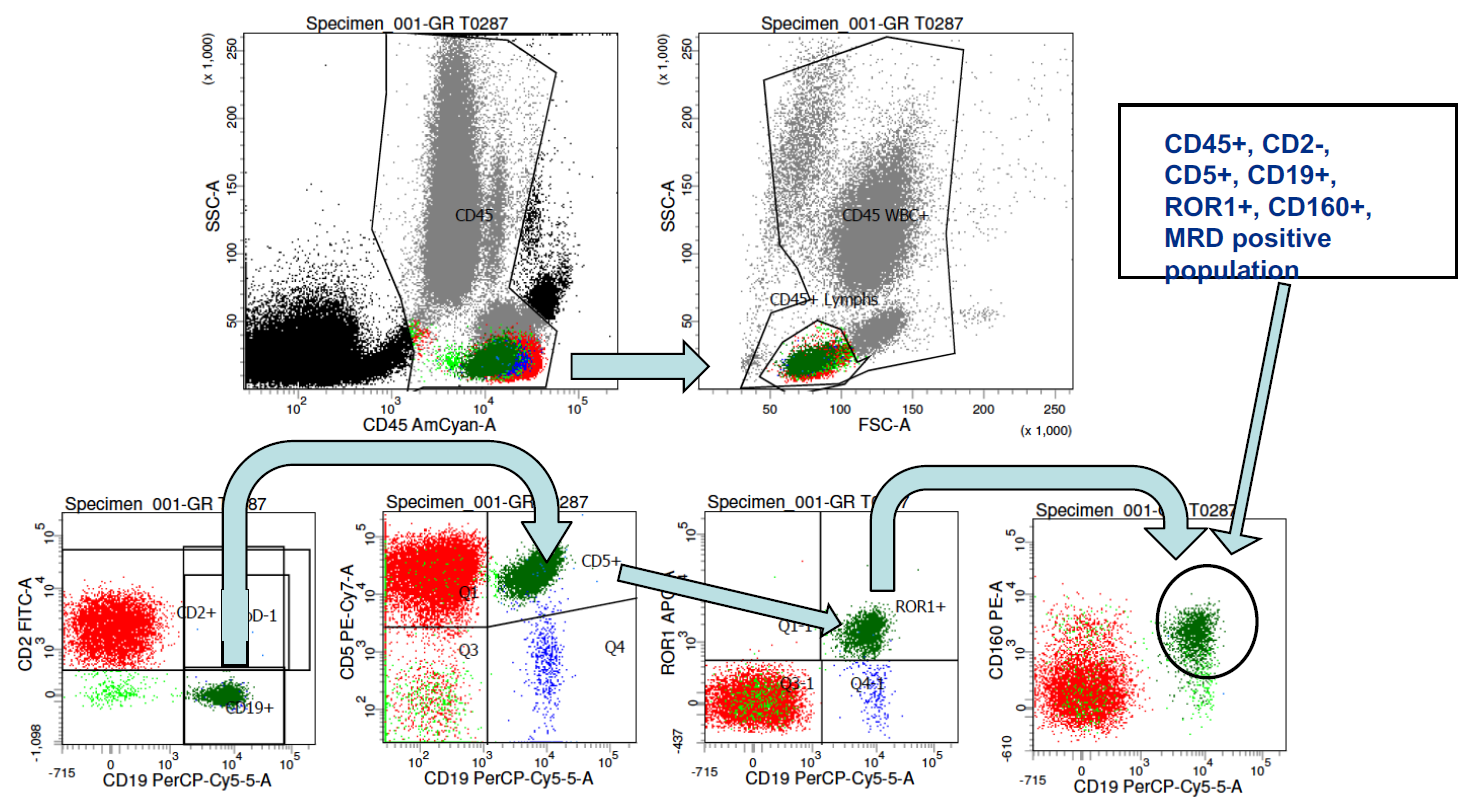


**Supplementary Figure 1: Gating strategy for the CD160-ROR1FCA.** Identification of residual disease populations used a validated sequential gating strategy for the detection of CD160 and ROR-1 co-expression on malignant CD19+ B cells. Initial gating focused on CD45 positive events versus side scatter, followed by forward and side scatter to gate the lymphoid region and exclude any apoptotic cells and debris. Total B cells were identified using CD19+ B cells were compared with side scatter to exclude any nonspecific binding.

**Supplementary Figure 2: CLL-like MBL specific expression of CD160FCA, the ERIC protocol and CD160-ROR1FCA.** CLL-like MBL cases and non-CLL like samples were assessed using the three assays. All three assays showed disease-specific antigen expression on the lymphoid population of CLL-like MBL patients, versus non-CLL like malignant cases (p < 0.01).
